# ‘It Shouldn’t Be This Hard’: Exploring the Challenges of Rural Health Research

**DOI:** 10.3390/ijerph16234643

**Published:** 2019-11-22

**Authors:** Heath Greville, Emma Haynes, Robin Kagie, Sandra C Thompson

**Affiliations:** 1 Western Australian Centre for Rural Health, The University of Western Australia, 35 Stirling Highway, Perth 6009, Western Australia, Australia; heath.greville@uwa.edu.au (H.G.); emma.haynes@uwa.edu.au (E.H.); robin.kagie@uwa.edu.au (R.K.); 2 School of Population and Global Health, The University of Western Australia, 35 Stirling Highway, Perth 6009, Western Australia, Australia; 3 Western Australian Centre for Rural Health, The University of Western Australia, 167 Fitzgerald St, Geraldton 6530, Australia

**Keywords:** rural, research, health services, ethics, governance, site-specific approval, Aboriginal

## Abstract

Health research is important for innovation and assessment of health status and health interventions, and maintaining a strong, engaged cohort of rural health researchers is essential for the ongoing improvement of the health of rural populations. Ethical guidelines and processes ensure research is undertaken in a way that protects and, where possible, empowers participants. We set out to systematically examine and document the challenges posed by ethics and governance processes for rural health researchers in Western Australia (WA) and the impact on the research undertaken. In this qualitative study, fifteen WA-based rural health researchers were interviewed. The identified challenges included inefficient systems, gatekeeping, apparent resistance to research and the lack of research experience of those involved in approval processes. For researchers seeking to conduct studies to improve rural and Aboriginal health, extended delays in approvals can hold up and impede research, ultimately changing the nature of the research undertaken and constraining the willingness of practitioners and researchers to undertake health research. Unwieldy ethics processes were seen to have a particularly onerous impact on rural research pertaining to service delivery, multiple sites, and research involving Aboriginal people, impeding innovation and inquiry in areas where it is much needed.

## 1. Introduction

Research is acknowledged as important for its contribution to building Australia’s innovation, productivity and growth, often with an emphasis on research–industry collaboration and the commercialisation of ideas. Rural health research would rarely have a commercial emphasis, but it is also recognised that Australia needs ‘better models of health care and services that improve outcomes, reduce disparities for disadvantaged and vulnerable groups, increase efficiency and provide greater value for a given expenditure’ [1]. A ‘strong research culture’ is needed to achieve this, and this requires understanding and embracing research as a way to improve health, ideally creating a partnership approach between researchers and end-users of the research [2].

Western Australia (WA) is Australia’s largest state, with a land area of 2,529,875 square km and occupying the entire western third of Australia. Three quarters of WA’s population lives in Perth with four remote regions of the state (Kimberley, Pilbara, Midwest-Gascoyne and Goldfields-Esperance) accounting for less than 10% of the state’s population. The low population density and large distances in rural WA create challenges for health service delivery, and the health status of people living in rural areas is known to be poorer [3]. Aboriginal people make up 4% of WA’s population; less than 5% of WA’s Aboriginal population live in Perth and the Southwest regions of WA, with high proportions in the Kimberley (over 40%), the Pilbara (15%), Midwest-Gascoyne (11%) and Goldfields-Esperance (10%) [4]. Aboriginal people continue to suffer from the consequences of colonisation and experience widespread socioeconomic disadvantage and health inequality [5].

Australia has a requirement that all research proposals involving human participants are reviewed by a Human Research Ethics Committee (HREC) to ensure they are ethically acceptable. Codes of ethics have evolved over time, as have institutional responsibilities for oversight of the conduct of research. Over 200 HRECs are now registered with the National Health and Medical Research Council (NHMRC), which provides guidelines for ethical research and has requirements for HRECs’ establishment, operation and membership. The need for ethical review and adherence to ethical processes is well accepted by researchers [5]. However, many researchers have previously detailed the cumbersome and lengthy processes of ethics and governance review and their impact on research in Australia [6,7,8,9,10], with many making constructive suggestions for how improvements could be made. There have been multiple calls for reform and streamlining of ethics approval and governance processes. We were interested to explore and document the views and experiences of rural health researchers and issues they encountered as they negotiated the ethics and governance approval processes required for research to be conducted.

### Theoretical Approach

The failure to adopt constructive suggestions for the reform of ethics and governance approval processes raises concerns that important social science research ‘that is both ethically and methodologically sound is being blocked and even stigmatised by the research ethics bureaucracy’ [11]. In a context where the value of public health/social research knowledge and the processes and methods for gaining such knowledge is contested, it may be helpful to conceptualise ‘power’ as a quality of social and institutional relations interacting with and determining the conditions of possible knowledge [12]. That is, recognising the institutional, cultural, political and economic influences on research agendas and methodologies suggests that there is a conflict between individualised biomedical research and community-orientated research that seeks to answer the ‘how’ and ‘why’ of public health issues.

## 2. Methods

In this qualitative study, fifteen WA-based health researchers who were undertaking research in a rural setting or with rural services were individually interviewed between 2014 and 2019. Participants were selected from a range of academic and research institutions. In addition to formal interviews, the researchers were informed by multiple conversations with colleagues reflecting their own engagement in rural health research, and notes were taken at a research forum of around 20 health researchers based in rural academic centres in Western Australia in 2019. Participants were recruited purposively initially and through snowballing as other potential participants were recommended.

A semi-structured interview guide (Appendix A) enabled individuals to ‘tell their story’ in a naturalistic way [13], capturing their emotional reactions and the impact of experiences on their motivation to conduct rural research in the future. Individual participants provided informed consent and were interviewed in person or by phone and digital recordings were transcribed verbatim. A standard qualitative research approach was employed to analyse the data. This included immersion in the data through multiple reading of the transcripts with annotations; identifying a thematic framework; coding and categorisation of emerging themes (undertaken independently by two team members who then synthesised and interpreted the themes); and refinement of the identification of themes in discussion among the research team with reference back to transcripts [13].

The research was approved in 2014 by the Human Research Ethics Committee of the University of Western Australia (RA/4/1/6860).

## 3. Findings and Discussion

Most of the researchers who were interviewed were based rurally, although five were based in the metropolitan area and involved in research with a substantial rural and Aboriginal component. The fifteen participants included four health professionals undertaking research in regional hospitals and health services for the purposes of quality improvement, and eleven researchers seeking to evaluate services, validate processes and identify best practice models of service delivery in regional WA, often including comparisons with other jurisdictions. Levels of experience ranged from early career researchers with less than 5 years’ experience of research (3 individuals) to two individuals with more than 25 years of research experience. Four interviews were undertaken in 2014 and eleven in 2018. The second round of interviews in 2018 aimed to capture the experiences of researchers following the introduction of new ethics and governance protocols in 2017.

Four key themes emerged: burdensome governance and site-specific approvals (SSA); complex and confusing multi-site approvals; unsuitability of governance and ethics processes for service evaluations and quality assurance; and delays and processes related to Aboriginal approvals.

### 3.1. Reported Additional Challenges for Rural Researchers: Governance and Site-Specific Approvals

It was clear in the early stages of our research that the nature of health issues investigated in rural research raised additional challenges for rural researchers—particularly if researchers were examining systems of care or transfers between an urban-based tertiary hospital and a regional hospital or clinic. Each site and region required its own governance or site-specific approval (SSA), requiring liaison with multiple Research Governance Offices (RGO). In regional settings, many of these positions were part-time roles, often not replaced when the person in the role goes on leave so that it was time-consuming to make contact with relevant individuals. One regionally-based researcher described a study in the Kimberley that required 22 separate site-specific approvals, with all 22 requiring final sign-off by the same person.

Regional decision-making bodies often wanted researchers to present health services research proposals and findings to a local health planning forum. This was an additional cost to researchers in time and travel, particularly if, in order to have sufficient numbers to power a study for intervention research, multiple regions were involved. This could require flying to several different parts of the state with flight times of up to three hours. These forums occur at different time frequencies and are often postponed or rescheduled, and in some regions held in different places. It was difficult for researchers to maintain a personal relationship with the service providers in multiple regions, particularly in rural areas because of workforce turnover. All delays meant that the time of research staff, employed to undertake the research, was spent getting the approval processes in place and completing forms that were described as long and repetitive, rather than progressing with data collection and analysis. The processes in fact reduced the opportunities for service engagement and ultimately feedback about the research.

Between 2014 and 2018, the WA Department of Health instituted an electronic Research Governance Service (RGS) to manage the WA Health Single Ethical Review and National Mutual Acceptance processes as well as the separate Research Governance process (https://rgs.health.wa.gov.au/Pages/Research-Governance.aspx). Several of the interviewees had experience of the RGS during 2017 and 2018. While generally supportive in principle of the move to a single on-line ethics application process, interviewees reported that the RGS has become a ‘new nightmare’. They reported clunky, inefficient software that requires choreographed input steps between the principal and other investigators, and opaque systems and instructions, which were absent or very difficult to understand, with support staff who often struggled to answer questions.

*The RGS system was a complete nightmare. You couldn’t move from one page to the next unless you had a specific thing clicked and at one point we were kind of joking. Between the three of us we had 10 undergraduate and post graduate degrees and none of us could work it out…We started back in February. I had two projects that started almost simultaneously with two junior staff who have time restrictions whilst they’re with us, so you want to be able to achieve things. So about 7 months later we got approval. And that was after hundreds of hours of inefficient nonsense that had nothing to do with the core component of the application and everything to do with getting through the system*.

(P8)

Another researcher commented

*I like the idea of a centralised system…. I’m all for computerised documentation. However, it was terribly inefficient, there were a lot of bugs in the system that resulted in a lot of man-hours wasted. It delayed publication, presentation and data collection. There were at least two upgrades while I was using the system. It shouldn’t have been on-line before it was properly tested. … It was the unfriendliness of the system that was awful*.

(P10)

Researchers found that the RGS was designed for clinical trials and intervention research that needed adequate safeguards and protection of participants from unproven treatments, with the controls and questions not appropriate for health service evaluation or quality assurance.

*The software needs to be more intuitive because the fields don’t match with research that’s not clinical trials or drug interventions. So that immediately puts qualitative researchers, social science researchers—all of which seem to be more prominent in rural areas—at a disadvantage. It’s not fit for purpose for rural researchers*.

(P4)

One researcher led a team conducting a small but important validation study in WA hospitals. The outcome of the research would ultimately benefit patients being treated for heart attack. All ethics reviews had been completed, all hospital CEOs had given individual approval and the team was ready to collect the data. However, the study had to abandon data collection from the three rural hospitals when, after three months of attempting to achieve them, site-specific approvals had barely begun.

*I find research governance now to be—and I know I’m speaking harshly—something that is designed to block research…After three months we said to ourselves ‘We just can’t do this anymore’. So we had to drop those rural hospitals…The outcome meant we couldn’t get the rural data. So we had many fewer Aboriginal patients and no rural patients*.

(P13)

Another researcher was part of a team attempting to determine whether patient wishes were followed at end of life through advance care planning. Twelve months of a two-year grant were taken up with ethics and governance approval.

*It’s…three months of fierce productive work to get the protocol ready, that’s sort of understandable...But then nine months of bouncing between different committees along with this administrative side of things*.

(P4)

This participant’s experience was reiterated by many interviewees. While they appreciated any help offered, researchers commented that the governance process and officers in the RGO were distant from the researchers, often poorly informed, gave conflicting advice or were often only able to provide advice on a small part of the process.

*There was conflicting information given by different officers on different sites which was very damaging because that sent us down different paths that we then found out were wrong. We wasted all this time going and getting the work done and there didn’t seem to be any one document that anyone said, ‘this is the thing to use...’Where there were guidelines, they were unclear or contradictory … and they didn’t say which one sort of trumped which, or was the most important thing to follow. …. It’s inefficient at both ends—researchers unfamiliar with each hospital’s processes are wasting their time and the hospitals’ time making endless phone calls, asking around and following up*.

(P4)

While some interviewees found RGO staff willing to be helpful, others had less positive experiences and reported antagonistic responses which obstructed the progress.

*(The governance process and the people running it) need to realise that research is important. It’s funded by public money—mostly by the NHMRC (National Health and Medical Research Council) and sometimes by Department of Health itself…It needs to be a two-way. Sure, you need to have something there to protect the institution…But they must also understand that research has to take place because there is a public benefit and patient benefit from research. We’re using public money so we can’t waste it*.

(P13)

### 3.2. Multi-Site Approvals and Reporting

Despite efforts to streamline multi-site processes through NHMRC-certified Ethics Committees, cross-jurisdictional approvals were reported as increasing the confusion. Interviewees engaged in multi-site research across Australian states and territories reported that some HREC (Human Research Ethics Committee) administrators were unaware of the extent of their own committee’s jurisdiction or of the interface between their HREC approval and SSAs. Processes differed between states and territories and when changes were introduced, it was not clear to everyone involved. Researchers were given conflicting information, and it was unclear where to apply for ethics review and for site-specific approval, again leading to waste of time by researchers and those tasked with administration of approvals in RGOs and health services.

Another source of frustration and inefficiency reported was the lack of standardisation in reporting requirements. Each ethics committee was reported as having different requirements related to frequency and the nature of reporting required and whether a reminder of the report being due is sent—an issue which has been previously reported [9]. This was highlighted by one interviewee who was administering the reports for a multi-site project across several states and territories.

*There have been up to 19 reports required. Some reports are for state-wide committees, some are for district level and some are site-specific. Each site or HREC has different reporting forms, different dates and different requirements. Some are very simple 1–2 page documents, while others are 7–9 pages. Many forms are designed for a clinical trial, not for service evaluation or QA (quality assurance)-type research. The forms are often completely unsuitable*.

(P12)

### 3.3. Service Evaluations and Quality Assurance

The patient journey is often more complex for rural patients, particularly when the required acute care is only available in a tertiary hospital based in the metropolitan area. Health services research, which focusses on systems of care beyond a single institution or region, offers the potential to identify where improvements around patient care can be made, but progressing such research was seen as increasingly difficult.

Participants commonly reported that processes are inappropriate for low-risk quality assurance studies and evaluations, which seek ethics approval in order to be published. While the University ethical approval process allowed for a ‘low-risk’ option, this was not available through either RGS or Aboriginal specific ethics. Moreover, research that was seen to specifically recruit Aboriginal participants was regarded as “high risk” and was required to go through an additional ethics approval process (elaborated in Section 3.4).

Many participants noted that questions and fields in the RGS and other HREC applications do not match the parameters of qualitative, health service improvement research. Processes were seen as out of proportion to the size of the study and the number of participants:

There’s no sense of proportionality…It seems heavily weighted towards large scale clinical trials where you can afford to spend a few months getting approvals in order to have access to hundreds of extra patients or whatever is your unit of examination…

(P4)

The ethics and governance approval processes were widely regarded as deterring rural researchers and those who wish to do research that can benefit rural populations.

*I’ve looked at processes in other states and I’ve considered moving to Sydney because it works well there—the processes have been streamlined. I’m not making the best use of my time here. I’m bewildered by this process*.

(P4)

Researchers commented upon research as an open process and subject to scrutiny but that current processes also meant that there was censorship of how research could be presented. There was a belief that research that examines service quality should be explicitly supported.

### 3.4. Aboriginal Research Approval Processes

Aboriginal-focussed research or research in Western Australia that is likely to include a reasonable proportion of Aboriginal people is required to be approved by an additional research committee, the Western Australian Aboriginal Health Ethics Committee (WAAHEC) to help ensure that health research is sensitive to Aboriginal culture and people. It is important that research in this context is undertaken respectfully and that publications avoid a pejorative and deficit representation of Aboriginal people. In its documentation, WAAHEC commits to communicate reasons for rejection of a project and gives applicants the opportunity to attend a WAAHEC meeting to present/defend their application. Most of the researchers interviewed reported positive experiences with the WAAHEC in terms of responsiveness. The process of working through the questions relating to Aboriginal cultural values was generally considered valuable in thinking through the broader ramifications and context of the proposed research, although two participants noted that the questions were not applicable to quality assurance studies.

While generally supporting the process of obtaining separate ethics approval from the WAAHEC, researchers noted that the additional process can require considerable extra time. Requirements for presentation of the research at local regional planning forums and for a letter of support from local Aboriginal organisations led to extra costs and delays. In particular, this made the difference for small projects when one or two extra months of waiting made the project unviable. It reflects the limited number of times the regional forums and Ethics Committee meet and that occasionally meetings are cancelled or postponed.

One researcher proposed conducting an evaluation of an adjunct program in a rural mental health service. Ethics approval was needed in order to publish the results.

*We were on a pretty tight timeframe. Initially, we wanted to get the ethics done within six months and because we were going through the new RGS, it had already taken us so much time. We foresaw that two more months to get approval from the WAAHEC was going to blow it out. And the other person who was starting was a registrar, and she was only with us for another six months, and it became completely unacceptable for that kind of time delay. Ultimately, we could still see the data and it’s our service, we knew what we needed to do. We could see the Aboriginal status. What we can’t do is use the data for publication purposes. Our study is not ground breaking. But it is relevant to other rural services—how they might want to set it up…The impact is small but it is a pity we weren’t able to publish the data and help Aboriginal young people*.

(P8)

WAAHEC wants to ensure that research is supported by the local community and avoid unnecessary burden on Aboriginal people and communities. The need for local Aboriginal understanding of and input into research was not disputed, as well as the right of those Aboriginal people directly involved to reject research proposals that are inappropriate for local circumstances and/or are culturally insensitive. Participants acknowledged past unethical practices in undertaking research that were exploitative and disrespectful of Aboriginal people and supported steps to address that occurring.

*I’m upbeat and positive about the WAAHEC process and the way the WAAHEC is aligning ethics approval with community approval and requiring that researchers seek approval from the communities and deliver benefits back to communities… those sort of values really fit with what I hope that ethics review would be about*.

(P4)

While researchers upheld the principle of local approval and guidance, some researchers reported that the requirement for a letter of support from an Aboriginal Community Controlled Health Organisation (ACCHO) created an unreasonable impediment to research progressing. Examples were provided of considerable engagement on the ground with local Aboriginal organisations and community members who were supportive of the research going ahead, but where the ACCHO was not working in that location or involved with the community or in the initiative. A letter signed by the ACCHO Chief Executive Officer (CEO) or Chair was seen to be highly dependent on individual personal relationships. A relationship-based system of approval means that ‘community approval’—potentially a process of discussion within a group or between leaders—can fall to the decision of one individual. Moreover, participants described challenges in meeting with the relevant person, cancelled meetings and a lack of response that could lead to long delays, often with no explanation. The local approval process can become one of gatekeeping, which stifles research rather than safeguarding the rights of patients and communities [14].

*We tried and tried but we never got a response. The research would have been valuable to the people of the region but somebody took a set against it for some reason and there was nothing we could do. There is no recourse when unethical processes stall progress*.

(P1)

The sometimes lengthy process of obtaining a local letter of support meant that smaller studies could not proceed or quality improvement studies could not be published because the time it took to get Aboriginal ethics approval blew out the timelines. One researcher reflected on the impact on smaller projects of the time it takes to build local relationships:

*Working in Aboriginal health research, we know its very relationship based, we know extra time is needed and so on, to work up research…But without a massive amount of resourcing, which is getting increasingly hard…it makes it difficult...If you had a big research grant, you’d be wanting to spend a lot of time in all of your sites but it’s just not possible without a big NHMRC project or something like that*.

(P9)

In a different region of the state, another research project was not given approval by the local research committee to enable Aboriginal interviewees to have input into the evaluation of a new chronic disease servicing arrangement but the reasons were not made clear.

*…the result of not including (this region) will lead to less Aboriginal control for the people of (the region) because they can’t have a say on how well or poorly the service is doing*.

(P6)

Problems with obtaining local support, including unanticipated difficulties with consultation processes and barriers to travel to establish face-to-face relationships have been described by McLoughlin and colleagues [15]. They suggested that institutional timeframes and funding schedules are part of the problem and that they undermine the aims of ethical guidelines and perpetuate the dominance of non-Indigenous control over the research agenda.

An evaluation of two key documents providing guidance for researchers undertaking research with Aboriginal people (Guidelines for Ethical Conduct in Aboriginal and Torres Strait Islander Health Research [16] and Keeping Research on Track: A Guide for Aboriginal and Torres Strait Islander Peoples About Health Research Ethics 2005 [17]) found that amongst other issues, peak bodies reported being overwhelmed with having to manage the volume of proposals but also that ‘despite the difficulties encountered in the research process, communities did not want researchers and HRECs to see Aboriginal health research as too hard, rather that there are processes that can be followed that will improve the research quality and output’ [18]. However, as researchers in this study commented, the time spent writing and rewriting ethics applications to meet multiple ethics requirements and doing SSAs is in fact counter-intuitive to better engagement with communities on the ground and ultimately leads to less time and opportunity during and at the end of the research for feedback to the participants and communities involved. As noted by Haynes et al. ‘a tendency for HRECs to rigidly pursue adherence rather than seeking to apply the spirit of “guidelines” can become another colonising practice’ [19].

Our study highlights that inefficient and unnecessarily complex ethical clearance processes lead to ‘research fatigue’ in communities and reduces the accountability of services and decision makers. Imposing a one-size-fits-all approval regime, including for low-risk research, works against the interests of improving Aboriginal health. Time-consuming approval processes for low-risk studies mean that researchers may decide not to investigate the appropriateness of services for Aboriginal people as a sub-group, or to stratify their results by Aboriginality. Opportunities to find out what works or doesn’t work for Aboriginal patients are lost. Findings and insights that can improve services and health outcomes for Aboriginal people are not documented and shared.

There is an argument that Aboriginal health ethics proposals should have an approval process for low-risk research. Streamlining the local approval processes by benchmarking and tracking response times could also make research more accountable for local communities.

The four themes are summarised in Table 1 below.

Researchers are often committed to developing new knowledge, which can inform understanding and service improvement. They are very often undertaking research on top of their service and clinical commitments because they see the need for service improvement and attention on underserved populations and new and better ways of doing things. While such attention should be encouraged and supported, many rural researchers feel that they are not able to influence ethics processes that are beginning to stifle research.

## 4. Conclusions

Our study set out to describe the particular challenges of meeting the requirements of ethical review and research governance experienced by those undertaking rural research, many of whom focus on health equity issues and how health and health care can be improved for people living in rural settings.

Rural researchers in Western Australia were often dealing with systems that involved multiple health services and Aboriginal people, increasing the burden associated with approvals. Ethics review and SSA were holding up smaller projects, quality assurance and validation studies, stifling rural research and deterring young researchers from entering into rural and Aboriginal research. More experienced researchers were reconsidering the nature of research they would undertake. The participants described the additional demands, challenges and costs they experience undertaking research, and which occur in the face of rural research being underfunded [20]. Participants in 2018 overwhelmingly cited frustrations in trying to navigate a new state-wide system developed around research governance in WA Department of Health sites.

Our findings highlight that, despite the stated efforts to streamline them, inefficient ethics review and governance processes are continuing to hinder the development of a strong research culture in WA. As described by other researchers [7], site-specific assessment has eclipsed ethical approval as the major holdup for research projects. A culture of ‘us and them’ was seen to exist between frustrated researchers and RGO officers who themselves struggle to understand procedures, which vary from site to site and from jurisdiction to jurisdiction. Most importantly, these processes waste valuable health research funds that should be giving direct benefit to the Australian population [21], as well as the time and energy of those working towards better knowledge, understanding and system improvement. As has been recommended by others, there should be a clear process and pathway to differentiate what constitutes ‘low-risk’ research, and to track ethics and governance processes from initiation to approval and benchmark them [22] as has been proposed as part of HREC certification [23].

Our study has identified some of the circumstances where ethics and governance overload is counterproductive to achieving research outcomes, and that this may particularly affect disempowered, disenfranchised populations [24]. We argue that this is an outcome of extending a medical model of research ethics to non-medical research without considering what might really be needed [25], and that ‘this trend portends a form of colonization by the medical sciences at the expense of the culture of the social sciences’ [26].

The proposal of Clay-Williams and colleagues for a single, centralised national system for all ethics and governance review processes appropriate for non-clinical research, which would allow researchers to apply for, amend and report on multi-centre studies, demands action. Such a system should be supported with resources and ongoing training for HRECs and RGO staff about health services research, including the way research is funded. There is an argument for such positions to be filled by individuals with experience of undertaking research. Given the dominance of the biomedical model in approval forms and processes, those with a real understanding of bioethics and with experience with qualitative, social and public health research could add value. Any centralised system needs to be as simple as possible, carefully planned and thoroughly tested, with adequate staff training before it is unleashed on the research community. In the meantime, and as a matter of urgency, Departments of Health should simplify the processes of SSA and the NHMRC should ensure that SSA processes are harmonised across the country.

Research and evaluation are important to highlight health disparities and to examine ways in which health and health service delivery can be improved. For rural populations that experience a higher burden of ill-health, investigating, evaluating and improving health systems is critical. However, the impediments to applying a critical lens to systems, programs and service delivery are exacerbated by distance, by funding and by the tortuous state of ethics and governance processes this study has described.

## Figures and Tables

**Table 1:** Reported additional challenges for rural researchers.

Themes and Issues	Example of Issues Raised
1. Governance and site-specific approvals Delays blow out budgets and timelines Time-consuming governance processes including documenting and costing staff time to the minute High staff turnover in regions; Repetitive processes; Poorly designed systems; Poorly trained support staff; Gatekeeping; Adversarial “us and them” culture.	*We were on a pretty tight timeframe. Initially, we wanted to get the ethics done within six months and because we were going through the new RGS, it had already taken us so much time. We foresaw that two more months was going to blow it out. And the other person who was starting was a registrar, and she was only with us for another six months, and it became completely unacceptable for that kind of time delay*. (P8) *I’d describe the experience as terrible and unnecessarily complicated. My research associate described the people she was dealing with (in the RGO) as ‘almost militant’. Like they have the power over us, that kind of attitude. That is absolutely unacceptable in medical research and it just has to change. And it seems to me that governance believes they are above everyone else and they control everything. If they say ‘no’ then you can’t do anything. And it cannot be like that in research…*(P13)
2. Multi-site approvals and reporting Process of nationally certified ECs appear not well understood by the certified committees themselves; Reciprocal processes uneven between committees and regions/jurisdictions; Onerous reporting to multiple committees on different time schedules; Complexity of the process discourages ambitious projects that deliver more generalisable results. Processes change without this being clear to rural researchers.	*(In the future) I wouldn’t try to engage with so many sites. I’d encourage smaller, less ambitious projects…You could do a small one in one community and have something to give back to that community. But it’s a bit more ambitious to have input into health planning more generally. Multiple sites make more sense from that perspective. When you’re talking about regional Australia, you can’t generalise from one area so you need more diversity in what you’re looking at…it’s all about diversity and small projects just don’t deliver on that. They can have good insights but service level projects need a broader brush.* (P15)
3. Service evaluations and quality assurance Committees don’t understand the nature of the research, especially qualitative studies and evaluation research; Forms and systems unsuitable for service research.	*The software (RGS) needs to be more intuitive. The fields don’t match with research that’s not clinical trials or drug interventions. So that immediately puts qualitative researchers, social science researchers…all of which seem to be more prominent in rural areas—at a disadvantage. It’s not fit for purpose for rural researchers*. (P4)
4. Aboriginal research approval processes Individuals can dominate the process; No low-risk pathway.	*The moment you mention “Aboriginal” research, the level of caution escalates. I can understand why but it’s ironical that in the service of improving health care for Aboriginal people, you can experience so many barriers. Of course, you want ethical processes and due diligence but I’m not sure that this process encourages good research*. (P12)

## Appendix A

### Interview Guide

- What areas of rural research are you predominantly involved in?
- Is there a particular project that best illustrates your experiences with the ethics process?
  - What was the purpose of this research? What were you trying to discover?
  - Did it have an Aboriginal focus?
  - Single site or multisite?
  - Was it qualitative and/or quantitative?
  - Which research partners were involved?
- What is your understanding of the role of the ethics approval process?
- What was your experience of the ethics application process?
  - What ethics approval processes were involved?
  - Which ethics committees did you apply to?
  - What levels of risk were associated with those applications?
  - What was your role in applying for ethics approval?
  - How would you describe your experience?
  - Did the ethics approval process improve the quality of your research or the process?
  - Did the application process pose any difficulties for you? Can you describe these?
  - To what extent do you think difficulties in the ethics process were associated with the research being Aboriginal/rural?
- Did the ethics approval process influence the kind of data/information you sought?
- Did the process stop you from analysing or publishing data?
- What has been the effect on the quality of the research produced? Improved?
- Have your experiences with the ethics approval process influenced your decisions to undertake future research?
- What positives do you think occurred because of the application process?
- Have you ever challenged an ethics committee about their process? What was the outcome?
- How do you regard the process after having completed it?
- What recommendations do you have about how the process could or should be changed? And why?
